# Supplementary material for: Proximate Drivers of Population-Level Lizard Gut Microbial Diversity: Impacts of Diet, Insularity, and Local Environment
Source: Microorganisms. 2022 Jul 31;10(8):1550. doi: 10.3390/microorganisms10081550 (PMC9413874; doi:10.3390/microorganisms10081550)
Supplement: Supplementary file 1 [file microorganisms-10-01550-s001.zip › Lemieux-Labont¿a et al Suppl Table S1 final.pdf]

Supplementary Table S1.

Each row is an individual sample. Indicated are the geographic origin of the sample, the diet (insect. = insectivorous; omn. = omnivorous), the sex (M = male; F = female), the year of sampling, the season, the number of 16S reads, the number of 16S reads after filtering, and the number of OTUs per sample.

**Lizard ID**	**Geographic origin**	**Diet**	**Sex**	**Year**	**Season**	**Number of 16S reads**	**Number of 16S reads after filtering**	**Number of OTUs**	**Genebank sample code**
PSKM34DIGC	Pod Kopište	insect.	M	2014	summer	66252	64162	2262	PSK34MDI
PSKM35DIGC	Pod Kopište	insect.	M	2014	summer	91720	89210	2062	PSK35MDI
PSKM36DIGC	Pod Kopište	insect.	M	2014	summer	60300	58479	2192	PSK36MDI
PSKM47DIGC	Pod Kopište	insect.	M	2016	summer	96401	93455	2544	PSK47DIGC
PSKM48DIGC	Pod Kopište	insect.	M	2016	summer	96206	93723	2202	PSK48DIGC
PSKM49DIGC	Pod Kopište	insect.	M	2016	summer	116439	114246	2290	PSK49DIGC
PSKM50DIGC	Pod Kopište	insect.	M	2016	summer	101185	99003	2160	PSK50DIGC
PSKF20DIGC	Pod Kopište	insect.	F	2014	summer	61099	59402	2322	PSKF20MDI
PSKF21DIGC	Pod Kopište	insect.	F	2014	summer	268851	261333	3350	PSKF21MDI
PSKF23DIGC	Pod Kopište	insect.	F	2014	summer	89080	86360	2997	PSKF23MDI
PSKF36DIGC	Pod Kopište	insect.	F	2016	summer	99245	97030	2402	PSKF36DIGC
PSKF37DIGC	Pod Kopište	insect.	F	2016	summer	105287	102295	2621	PSKF37DIGC
PSMM30DIGC	Pod Mrčaru	omn.	M	2014	summer	66288	64095	2622	PSM30MDI
PSMM31DIGC	Pod Mrčaru	omn.	M	2014	summer	51972	50329	2440	PSM31MDI
PSMM32DIGC	Pod Mrčaru	omn.	M	2014	summer	62676	60827	2418	PSM32MDI
PSMM33DIGC	Pod Mrčaru	omn.	M	2014	summer	40076	38712	1801	PSM33DIC
PSMM50DIGC	Pod Mrčaru	omn.	M	2016	summer	72679	71675	915	PSM50DIGC
PSMM51DIGC	Pod Mrčaru	omn.	M	2016	summer	69741	68336	1884	PSM51DIGC
PSMM52DIGC	Pod Mrčaru	omn.	M	2016	summer	57238	55478	2399	PSM52DIGC
PSMM53DIGC	Pod Mrčaru	omn.	M	2016	summer	54389	52709	2296	PSM53DIGC
PSMF18DIGC	Pod Mrčaru	omn.	F	2014	summer	57391	55488	2339	PSMF18MDI
PSMF19DIGC	Pod Mrčaru	omn.	F	2014	summer	167060	161837	3150	PSMF19MDI
PSMF20DIGC	Pod Mrčaru	omn.	F	2014	summer	57819	55901	2428	PSMF20MDI
PSMF32DIGC	Pod Mrčaru	omn.	F	2016	summer	94521	91570	2755	PSMF32DIGC
PSMF33DIGC	Pod Mrčaru	omn.	F	2016	summer	91263	88672	2681	PSMF33DIGC
PSSM20DIGC	continent	insect.	M	2014	summer	59615	57868	2280	PSS20MDI
PSSM21DIGC	continent	insect.	M	2014	summer	37356	36321	1791	PSS21MDI
PSSM22DIGC	continent	insect.	M	2014	summer	65503	63520	2058	PSS22MDI
PSZM31DIGC	continent	insect.	M	2014	summer	80577	78493	1947	PSZ31MDI
PSZM32DIGC	continent	insect.	M	2014	summer	53988	52246	2011	PSZ32MDI
PSZM33DIGC	continent	insect.	M	2014	summer	69702	67830	1554	PSZ33MDI
PSSM34DIGC	continent	insect.	M	2016	summer	74189	72017	2105	PSS34DIGC
PSSM35DIGC	continent	insect.	M	2016	summer	70318	68221	2219	PSS35DIGC
PSSM36DIGC	continent	insect.	M	2016	summer	92548	90419	2252	PSS36DIGC
PSSM37DIGC	continent	insect.	M	2016	summer	95826	92791	3079	PSS37DIGC
PSSF10DIGC	continent	insect.	F	2014	summer	55412	54033	1646	PSSF10MDI
PSSF8DIGC	continent	insect.	F	2014	summer	58646	56987	2137	PSSF8MDI
PSSF9DIGC	continent	insect.	F	2014	summer	59875	58136	1825	PSSF9MDI
PSSF22DIGC	continent	insect.	F	2016	summer	31664	30881	1761	PSSF22DIGC
PSSF23DIGC	continent	insect.	F	2016	summer	81199	79081	2078	PSSF23DIGC
