# Supplementary figures and images for: Proximate Drivers of Population-Level Lizard Gut Microbial Diversity: Impacts of Diet, Insularity, and Local Environment
Source: Microorganisms. 2022 Jul 31;10(8):1550. doi: 10.3390/microorganisms10081550 (PMC9413874; doi:10.3390/microorganisms10081550)

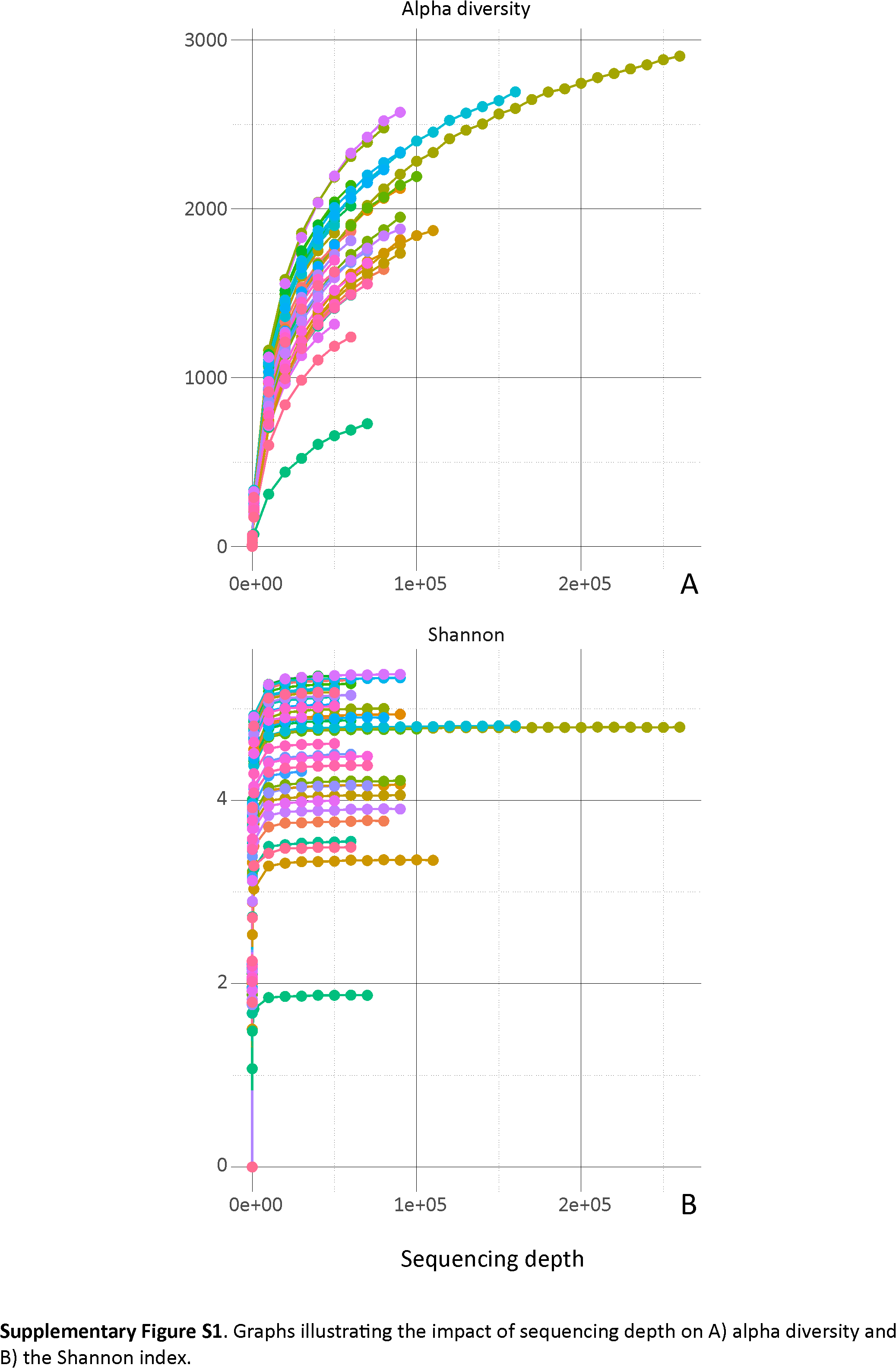

Supplement: Supplementary file 1 [file microorganisms-10-01550-s001.zip › figure S1.tif]

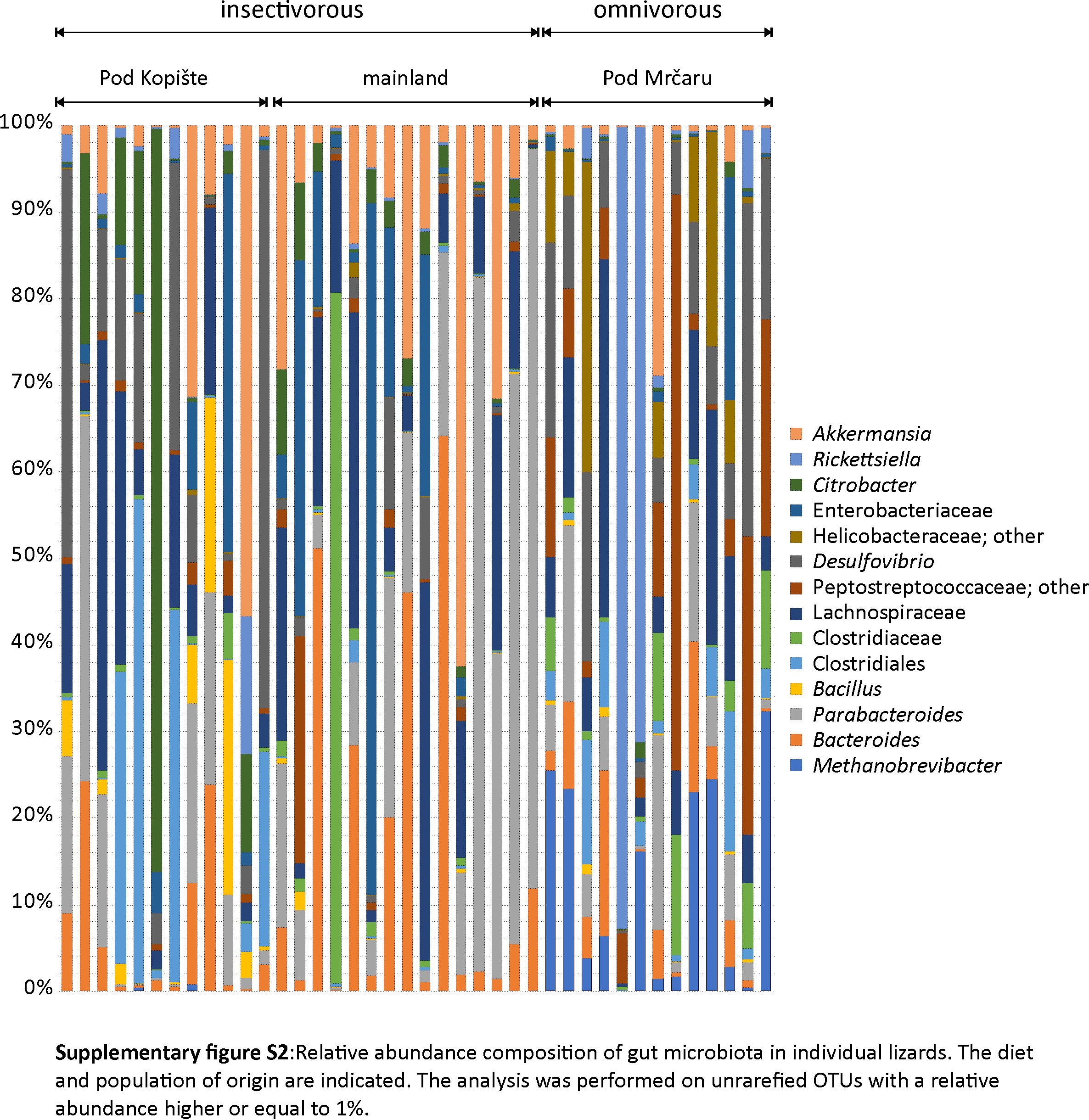

Supplement: Supplementary file 1 [file microorganisms-10-01550-s001.zip › figure S2.tif]

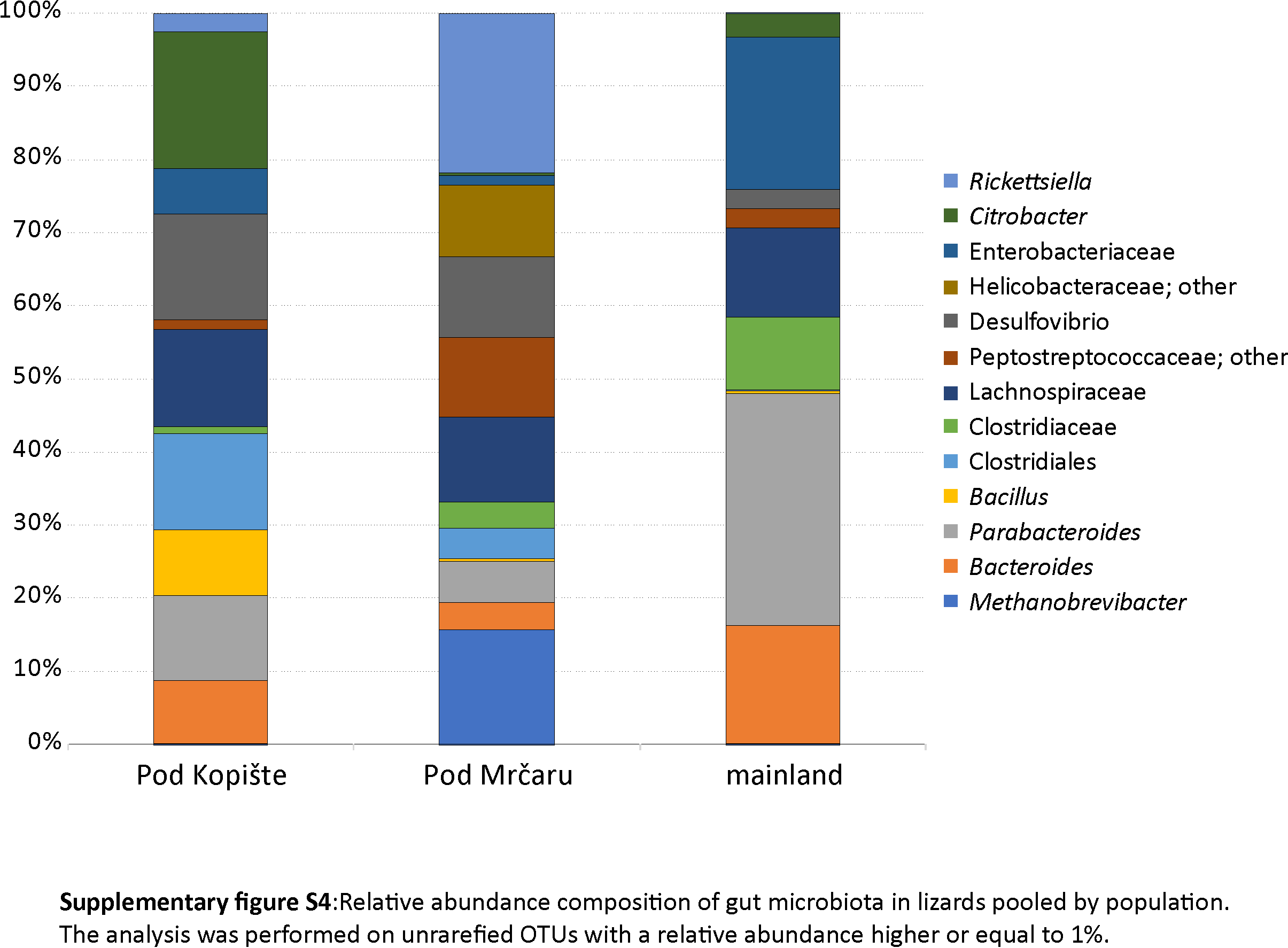

Supplement: Supplementary file 1 [file microorganisms-10-01550-s001.zip › figure S4.tif]
